# Effects of mindfulness on stress, life satisfaction, and savoring beliefs among Hong Kong Chinese adolescents during the COVID-19 pandemic

**DOI:** 10.3389/fpsyg.2023.1118288

**Published:** 2023-05-15

**Authors:** Ngar-sze Lau, Rebecca Y. M. Cheung, Cheuk Ki Stephanie Lai, Abby Yan Tung Lau, Man Ching Fung

**Affiliations:** ^1^Department of Educational Administration and Policy, The Chinese University of Hong Kong, Hong Kong, Hong Kong SAR, China; ^2^School of Psychology and Clinical Language Sciences, University of Reading, Reading, United Kingdom; ^3^Department of Special Education and Counseling, The Education University of Hong Kong, Hong Kong, Hong Kong SAR, China; ^4^Centre for Psychosocial Health, The Education University of Hong Kong, Hong Kong, Hong Kong SAR, China; ^5^The Jockey Club School of Public Health and Primary Care, Faculty of Medicine, The Chinese University of Hong Kong, Hong Kong, Hong Kong SAR, China

**Keywords:** wellbeing, mindfulness, stress, life satisfaction, savoring beliefs, adolescents, Hong Kong

## Abstract

Adolescents all over the world are vulnerable in facing developmental challenges. Recent studies have evidenced that the unexpected interruptions of school learning during the COVID-19 pandemic have raised concerns about the well-being of adolescents. This present study sought to investigate the relationship between mindfulness, stress, savoring beliefs, and satisfaction of life among adolescents in Hong Kong during COVID-19. A total of 240 Hong Kong Chinese adolescents between 15 and 19 years of age (M = 15.60; SD = 0.70) from schools with different religious backgrounds completed an online survey. Findings from hierarchical linear regression indicated that statistically, mindfulness negatively predicted stress and positively predicted life satisfaction and savoring beliefs. Students with faiths did not show any significant differences in mindfulness and other variables in this study from students without faiths. In terms of implications, these findings provide positive evidence that mindfulness may be an important aspect for interventions designed to enhance life satisfaction and savoring beliefs, and reduce stress of adolescents over challenging times. Overall, this study suggests youth service providers to develop effective strategies in schools and communities for further promoting wellbeing and resilience of adolescents.

## Introduction

Prior to COVID-19, studies found that factors such as unhealthy lifestyles, peer pressure, busy school schedules, and mood fluctuations were crucial for adolescents’ well-being (Lam and Hui, 2010; Neff and McGehee, 2010; Elgar et al., 2015). Since the declaration of the pandemic by the World Health Organization (WHO) in March 2020, the outbreak has had an unprecedented impact on the education, well-being, health, and mental health of youths due to school closures, home confinement, and social distancing policies (Rao and Fisher, 2021). It is estimated that 94% of children and youths were affected by the closures and 33% were unable to access remote online learning (UNICEF, 2021). Students from lower socioeconomic backgrounds, such as those with parents who had less education and those from areas with greater residential crowding, were more likely to be affected by school closures compared to their more advantaged peers (Rao and Fisher, 2021). As such, there is an urgent call to examine the needs of youths around the world (Rao and Fisher, 2021). The well-being and mental health of adolescents throughout the pandemic should also be given priority in education and social policies (Gore et al., 2011).

### Stress level and stress coping

The level of stress and coping of students during the pandemic has become a concern because unpredictability can affect the well-being, life satisfaction, and mental health of families and children (Moreno et al., 2020; Dymecka et al., 2021). A cross-sectional study by Evli and Şimşek (2022) found that mental distress, including anxiety, depression, and stress, emerged due to feelings of uncertainty and imaginary situations. Recent studies pointed out that adolescents were worried about their health situation (Li et al., 2022; Mikkelsen et al., 2022), relationships with classmates, such as social support (Larsen et al., 2022; Schoeps et al., 2022), and academic results (Lessard and Puhl, 2021; Tasso et al., 2021). A study by Rodríguez-Cano et al. (2022) revealed that anxiety over academic and economic consequences predicted adolescents’ poor psychological health, including poor emotion regulation and greater depressive symptoms, especially in families of adolescents with low socioeconomic status. Due to limited access to school counseling services, adolescents’ levels of stress and self-destructive behavior, such as self-injury, increased during the pandemic (Hasking et al., 2020; Orsolini et al., 2022). Therefore, assessing the stress level of adolescents for early intervention and exploring effective online stress coping strategies are extremely important.

### Well-being of adolescents

Life satisfaction is a component of subjective well-being (Pavot and Diener, 2008; Moksnes and Espnes, 2013). It is extensively evidenced that a high level of life satisfaction is related to physical and mental health. Life satisfaction is related to the quality of life of children and adolescents and is based on several factors, for example, social life, socioeconomic status, and affective experiences (Huebner, 2004). Adolescence is a critical period of cognitive, psychological, and physical development, and there is an increased level of stage-salient stress during this phase (Bergin et al., 2018). Several studies have found that life satisfaction is positively related to academic achievement (Diseth et al., 2012; Wong and Siu, 2017; Bozzato, 2020), whereas adolescents’ life satisfaction also influenced by the use of social media and peer relationships (Geraee et al., 2019; Orben et al., 2019). In the findings of a longitudinal study, adolescents who reported positive life satisfaction were at a lower risk of developing external behaviors in stressful events (Suldo and Huebner, 2004). Another large-scale study involving high-school students (*N* = 5,032) also found that life satisfaction was inversely related to alcohol and chemical use (Valois et al., 2001). Life satisfaction is also a negative predictor of suicidal ideation (Heisel and Flett, 2004).

It is evidenced that anti-epidemic measures, such as home confinement, decrease psychological health, as well as increase psychiatric symptoms among adults and adolescents (Rohde et al., 2020; Santini et al., 2020; Xie et al., 2021). Depressive symptoms are positively associated with home confinement. During the pandemic, adolescents faced various adjustments to their daily lives, for example, school closures, online learning, and missed extracurricular activities. Cross-sectional research by Schwartz-Mette et al. (2022) showed that the changes brought about by COVID-19 adversely impact adolescents’ depressive symptoms. Interestingly, research conducted by Sun et al. (2020) found that adolescents who had social support through social contacts were more likely to have fewer depressive symptoms during the pandemic. Thus, accessing the level of life satisfaction of adolescents can help determine their well-being and mental health risk.

Despite reports of the many negative effects of COVID-19 and the anti-pandemic policies, such as home confinement, some impacts have been neutral or positive. For instance, a US-based survey conducted during the pandemic found that people experienced high levels of parental warmth for their children and quality time with their children increased despite the hardships (Center for Translational Neuroscience, 2021). In another study, it was parental stress, instead of COVID-19 stress, that predicted parental burnout (Vaydich and Cheung, 2022). During this stressful period, it is important for people to be able to savor or derive pleasure from the past, present, and future. The process of savoring requires a mindful awareness of being conscious and enjoying the various experiences (Bryant and Veroff, 2007; Cheung and Lau, 2021). Based on previous research, a lower level of savoring is associated with hopelessness, depression, and anxiety (Bryant, 2003; Chiu et al., 2020). Therefore, identifying the level of savoring positive experiences may be helpful in accessing the potential needs of adolescents during the pandemic.

### Mindfulness, stress, life satisfaction, and savoring

Mindfulness has received a great deal of attention in the past three decades as an intervention for promoting well-being and preventing mental distress among clinical populations, general adults, and adolescents and children (Felver et al., 2016; Creswell, 2017). Mindfulness is defined as moment-to-moment awareness of the present moment, on purpose, and without judgment (Kabat-Zinn, 1990). Mindfulness is the capacity of self-regulation of attention so that it is maintained on immediate experience with curiosity, openness, and acceptance (Bishop et al., 2004).

There is a significant amount of empirical research supporting the idea that mindfulness is beneficial to well-being, including subjective feelings of happiness, life satisfaction, and positive emotions (Felver et al., 2016; Fabian, 2022). It is evidenced that mindfulness is a useful stress reduction strategy for adolescents (Lau and Hue, 2011; Felver et al., 2016). Apart from applying mindfulness to daily stressful life events, mindfulness also plays a crucial role in coping with traumatic events. Research has found that mindfulness practice favors the treatment of people who have experienced a disaster, showing that mindfulness is a protective strategy for stressful and advanced situations (Eriksen and Ditrich, 2015; Bergin and Pakenham, 2016).

Recent studies have revealed that there is a positive correlation between mindfulness and well-being (Hanley et al., 2014). It has been found that the positive relationship between mindfulness and well-being is mediated by self-esteem (Bajaj et al., 2016). Mindfulness is associated with life satisfaction mediated through the savoring of positive experiences and gratitude (Cheung and Lau, 2021). Another longitudinal study also indicated that mindfulness is associated with psychological distress *via* awareness and acceptance of negative emotions, impulse control, and emotion regulation (Cheung and Ng, 2019). A recent study involving Chinese adolescents demonstrated that mindfulness is not only positively associated with life satisfaction, self-esteem, and resilience, it also predicts life satisfaction through the mediating effect of self-esteem (Wang and Kong, 2020). Furthermore, in another study of Chinese adolescents, mindfulness was also found to enhance the meaning in life and life satisfaction as a mediating role (Dong and Geng, 2022). Overall, mindfulness not only cultivates an open and accepting awareness of one’s thoughts and feelings, it also facilitates life satisfaction through the savoring of positive experiences and meaning in life.

While the challenges faced by adolescents during the pandemic may vary from country to country, recent studies have shown that Hong Kong adolescents have experienced increased mental health risks, such as depression and anxiety, compared to the past decade (Lau et al., 2017; Ni et al., 2020). Investigation of the psychological condition of Hong Kong adolescents in the context of the COVID-19 pandemic is urgently needed and would be significant to society and the education sector in terms of exploring early intervention methods. However, there is a lack of research on the relationship between mindfulness, stress, and well-being among Chinese adolescents in the context of the COVID-19 pandemic. Hence, this study aims to examine these associations among Chinese adolescents in Hong Kong. The study hypothesizes that, first, mindfulness is negatively associated with stress; second, mindfulness is positively associated with life satisfaction; and third, mindfulness is positively associated with the savoring of positive experiences among Hong Kong Chinese adolescents during the pandemic. The research team also asked the adolescent participants a series of short questions to explore their religious affiliations, stress coping strategies, contemplation practices, and the duration of said practices.

## Methods

### Participants

The participants were 240 Chinese adolescents (52.50% boys, *n* = 126) recruited from three secondary schools in Hong Kong, ranging in age from 14 to 19 years (*M* = 15.60; *SD* = 0.70). The majority of participants reported that they were not affiliated with any religion (77.50%, *n* = 186). On a scale of 1 (not interested) to 4 (interested), participants reported that they were mildly interested in meditation (*M* = 2.80; *SD* = 0.78). A total of 76 participants reported that they had practiced meditation for 1 week or less, eight participants reported that they practiced for 2–3 weeks, 18 reported that they had practiced for 4 weeks or above, and 138 did not provide data on meditation practice. Regarding the duration of practice, 82 participants reported that each practice usually lasted 1–5 min, 13 reported that it usually lasted 6–10 min, five reported that it usually lasted 11–15 min, eight reported that it lasted 15 min or more, and 132 did not provide data on the duration of practice.

### Procedures

The research was approved in November 2020 by the Human Research Ethics Committee of The Education University of Hong Kong. Over the period of frequent school closures between December 2020 and February 2021, letters of invitation were sent out to the teachers and principals of secondary schools through snowball sampling, with brief information about the study and a sample questionnaire being provided. Three schools from different districts accepted the invitation and the research team invited senior secondary school students to voluntarily participate in this study from March to June 2021. As Form 5 and Form 6 students had tight school study schedules in preparation for their public examinations, only Form 4 students were targeted participants. The three schools had different backgrounds, i.e., Buddhist, Christian, and secular. A mixed-method research approach was implemented in this study. Consent was obtained from the schools and each participant before they were allowed to take the online questionnaire. At the end of the questionnaire, students were invited to attend a voluntary 15-min interview *via* an online social media tool.

## Measures

### Dispositional mindfulness

The Five Facet Mindfulness Questionnaire-Short Form (FFMQ-SF; Baer et al., 2006; Hou et al., 2014) was used to assess dispositional mindfulness. The measures comprised 20 items on five subscales: observing, describing, nonjudging, nonreacting, and acting with awareness. Participants rated on a five-point Likert scale ranging from 1 (*never/very rarely true*) to 5 (*very often/always true*). The raw scores were averaged, with higher averaged scores indicating greater mindfulness. Sample items included, “I pay attention to sensations such as the wind in my hair or sun on my face,” “In difficult situations, I can pause without immediately reacting,” and “When I do things, my mind wanders off and I’m easily distracted.” The Cronbach’s alpha for this study was 0.83.

### Perceived stress

The 10-item Perceived Stress Scale was used to access the perceived stress of the participants (PSS-10; Cohen and Williamson, 1988). Participants rated the frequency that they experienced each item on a five-point Likert scale ranging from 0 (*never*) to 4 (*very often*). Positive items were reverse scored and the item scores were then averaged, with a higher score indicating a greater level of perceived stress. Sample items included, “How often have you been upset because of something that happened unexpectedly,” “How often have you felt nervous and stressed,” and “How often have you felt difficulties were piling up so high that you could not overcome them?” The Cronbach’s alpha for this study was 0.67.

### Life satisfaction

Life satisfaction was assessed by the Satisfaction with Life Scale (SWLS; Diener et al., 1985), which is evidenced to be a reliable and valid measure for adolescents cross-culturally (Pavot and Diener, 2008). The scale comprises five items and ratings were given on a seven-point Likert scale ranging from 1 (*totally disagree*) to 7 (*totally agree*). The raw scores were averaged, with higher averaged scores indicating greater life satisfaction. Sample items included, “In most ways, my life is close to my ideal” and “I am satisfied with my life.” The Cronbach’s alpha for this study was = 0.83.

### Savoring

The 24-item Savoring Beliefs Inventory (SBI; Bryant, 2003) was used to assess perceived beliefs of savoring on an eight-item subscale that included anticipation, savoring the moment, and reminiscing. Sample items included, “Get pleasure from looking forward,” “Feel fully able to appreciate good things,” and “Easy to rekindle joy from happy memories.” Participants rated on a five-point Likert scale ranging from 1 (*strongly disagree*) to 5 (*strongly agree*). Item scores were averaged to form three subscale scores. Higher scores indicated a greater savoring tendency. The Cronbach’s alpha for this study was 0.78.

## Data analysis

### Quantitative analysis

Mean, standard deviation (SD), correlation, and hierarchical linear regression analyses were conducted using IBM SPSS Statistics 27. In the hierarchical regression models, demographic data including adolescents’ age, gender, and religion were entered in the first block as covariates. Adolescents’ mindfulness was then entered in the second block to predict perceived stress, savoring, and life satisfaction, respectively. Given that 48.75–57.50% of the data were missing for household income, meditation interest, meditation experience, and practices, the variables were not included as covariates in the regression analyses.

### Qualitative analysis

Online individual interviews with three adolescents were conducted. The transcripts were analyzed into several themes by content analysis, including sources of stress, coping strategies, interest in mindfulness, and mindfulness experiences.

## Results

### Quantitative findings

Table 1 indicates the mean, SD, and zero-order correlations of the variables. Table 2 indicates the findings from three hierarchical linear regression models, with adolescents’ demographic variables entered as covariates in Block 1 and mindfulness entered in Block 2 as a predictor of perceived stress, savoring, and life satisfaction. The first model with perceived stress as a dependent variable explained 14.98% of the variance in perceived stress, *F*(4, 235) = 10.78, *p* < 0.001. Notably, greater mindfulness was significantly associated with lower perceived stress among adolescents (β = −0.39, *p* < 0.001). The second model with savoring as a dependent variable explained 20.00% of the variance in savoring, *F*(4, 235) = 16.96, *p* < 0.001. Notably, greater mindfulness was significantly associated with greater savoring among adolescents (β = 0.45, *p* < 0.001). The final model with life satisfaction as a dependent variable explained 8.41% of the variance in life satisfaction, *F*(4, 235) = 7.16, *p* < 0.001. Notably, greater mindfulness was significantly associated with greater life satisfaction among adolescents (β = 0.29, *p* < 0.001). Furthermore, Table 3 indicates the findings of the reported usual practices of stress coping of the participants. Nearly half of the participants (46.7%) declared that the use of electronic devices, e.g., PlayStation, Switch, etc., was a common way of coping with stress. Nearly 80% of participants accessed YouTube to reduce their stress. Only a minority would reduce stress through sports activities (29.2%) and dancing (5%).

**Table 1:** Zero-order correlations, means, and *SD*s.

	1	2	3	4	5	6	7	8	9	10	11
1. Family income	—										
2. Age	0.13	—									
3. Gender (0 = male; 1 = female)	0.04	−0.16^*^	—								
4. Religion (0 = no; 1 = yes)	−0.06	−0.12	0.11	—							
5. Interest in meditation	−0.19	0.04	−0.10	−0.03	—						
6. Meditation experience (in weeks)	−0.25	0.11	−0.22^*^	0.03	0.16	—					
7. Minutes of practice per week	0.01	0.07	0.12	0.11	−0.21^*^	−0.06	—				
8. Mindfulness	0.04	0.11	0.03	−0.04	0.06	0.10	−0.10	—			
9. Perceived stress	0.03	−0.06	0.01	−0.03	−0.22^*^	0.02	0.11	−0.39^***^	—		
10. Savoring	−0.07	−0.08	0.13^*^	0.08	0.17	−0.15	−0.13	0.44^***^	−0.31^***^	—	
11. Life satisfaction	0.07	0.15^*^	0.02	0.02	0.10	−0.21^*^	−0.17	0.31^***^	−0.51^***^	0.35^***^	—
*Means*	1.58	15.60	—	—	2.80	1.63	2.79	3.03	3.01	3.34	3.89
*Standard deviations*	0.65	0.70	—	—	0.78	1.16	0.75	0.29	0.50	0.46	1.06

^*^p < 0.05. ^***^p < 0.001.

**Table 2:** Hierarchical regression models of mindfulness as a predictor of perceived stress, savoring, and life satisfaction among adolescents.

Variables	Stress				Savoring				Life satisfaction			
	Block 1		Block 2		Block 1		Block 2		Block 1		Block 2	
	β	*B* (*SE*)	β	*B* (*SE*)	β	*B* (*SE*)	β	*B* (*SE*)	β	*B* (*SE*)	β	*B* (*SE*)
Age	−0.06	−0.05 (0.05)	−0.02	−0.01 (0.04)	−0.05	−0.04 (0.04)	−0.11	−0.07 (0.04)	0.16^*^	0.24 (0.10)	0.12	0.19 (0.10)
Gender	0.01	0.01 (0.07)	0.03	0.02 (0.06)	0.11	0.10 (0.06)	0.09	0.08 (0.05)	0.04	0.09 (0.14)	0.03	0.06 (0.13)
Religion	−0.04	−0.05 (0.08)	−0.05	−0.06 (0.07)	0.06	0.07 (0.07)	0.08	0.08 (0.06)	0.04	0.09 (0.17)	0.05	0.11 (0.16)
Mindfulness			−0.39^***^	−0.67(0.10)			0.45^***^	0.72 (0.09)			0.29^***^	1.08 (0.23)
Adjusted *R*^2^	−0.01		0.14		0.01		0.21		0.01		0.09	
*R* ^2^	0.01		0.16		0.02		0.22		0.03		0.11	
*R*^2^ change	0.01		0.15		0.02		0.20		0.03		0.08	
D.f.	3/236		1/235		3/236		1/235		3/236		1/235	
*F* change	0.42		41.66^***^		1.93		60.58^***^		1.98		22.17^***^	

^*^p < 0.05; ^***^p < 0.001.

**Table 3:** Usual practices of stress coping among adolescents.

Usual practices of stress coping	Number	%	*N* (Total number of students who responded)
Dancing (hip hop, K-pop, Jazz, etc.)	12	5.0	240
Sports (football, basketball, running, etc.)	70	29.2	240
Electronic devices (play station, games, Switch, etc.)	112	46.7	240
Religious activities (visit churches)	3	1.3	240
YouTube (drama, music, etc.)	187	77.9	240
Others (reading, drawing, sleeping, playing with pets, etc.)	57	23.8	240

### Qualitative interview findings

From the interviews, two participants from the same school without a faith background expressed their interests and potential challenges. Pseudo names are used to protect the students’ identities. A male student, Gary, shared, “…I think mindfulness is training that can soothe the body and mind, and allows us to concentrate. In fact, I know that I am under pressure and those mindful practices are useful. I am curious to know and try it if I have time, as I know the benefits of mindfulness.” Gary expressed that he might follow mindfulness practices from social media and invite family members and friends to also practice.

A female student, Helen, said, “Sometimes when working on my assignments, I notice my heart beating fast… and once I could not sleep at night. When doing homework, I feel tense and anxious because my exams are soon, and I am weak in the subject.” She tried mediation in school and felt relief after the practices. “I felt relaxed psychologically. I want to train my patience and attention when eating and my attitude towards walking about at the same time. I often leave my seat and walk back and forth several times during dinner. I complain about being distracted and I really want to improve myself.”

Paul from the Buddhist school mentioned that his pressure and anxiety originated from his academic studies and family conflicts. Due to his bad mood and impulsive thoughts, Paul would practice mindfulness in his daily activities. “When taking a shower, I pay attention to the water flow and temperature in order to relax. Gradually, I have been able to maintain a certain level of attention through such practices.” Paul reported that the practices have changed his mental state, improving his attention span, emotional regulation, and even altruistic behavior. “I know that my classmates go to sleep late at night because they are on their phones. It is hard to relax [in this way]. I want to avoid looking at social media and using electronic gadgets late at night, so I choose to practice mindfulness, which works for me,” Paul added. The above cases show the feasibility of mindfulness practices for Chinese adolescents.

## Discussion

In this small-scale study involving 240 Chinese adolescents from three schools in Hong Kong, we investigated how mindfulness was related to stress reduction, life satisfaction, and savoring in light of the challenges posed by the COVID-19 pandemic, which has created a critical health and humanitarian crisis. The major findings show that mindfulness was inversely correlated to stress, but also predicted a lower level of stress in the regression analysis. Mindfulness, which cultivates moment-to-moment non-judgmental awareness, induces a non-reactive psychological change mechanism towards a negative environment. The results are consistent with the previous findings that mindfulness can act as a buffer towards the effects of perceived stress on depression and anxiety among adults (Bergin and Pakenham, 2016) and adolescents (Lau and Hue, 2011; Felver et al., 2016).

Regarding the well-being of the participants in this study, mindfulness predicted both life satisfaction and savoring in the regression analyses. Both life satisfaction and savoring were positively associated with each other, and negatively associated with stress. These results are consistent with previous research which finds that mindful awareness, with its function of openness and curiosity, enhances the capacity to savor and enjoy positive experiences in the past, present, and future (Bryant and Veroff, 2007; Cheung and Ng, 2020). Mindfulness was related to life satisfaction with the mediating role of savoring (Cheung and Lau, 2021). Mindfulness also predicted life satisfaction and meaning in life for Chinese adolescents (Dong and Geng, 2022).

Furthermore, from the questions asked about the usual practices of coping with stress, it was found that the majority of adolescents had high exposure to electronic devices and social media, which might have been due to the pandemic. Recent studies have argued that excess screen time among youths during the pandemic has resulted in eye discomfort, unhealthy eating habits, family conflicts, concentration difficulties when studying, and even mental health problems (Rao and Fisher, 2021; Ho and Lee, 2022). However, in other studies, the use of social media may increase the life satisfaction of some adolescents because it can enhance peer relationships, especially during school closures (Geraee et al., 2019; Orben et al., 2019).

From a previous study, adolescents with high levels of spiritual experience benefited from both religious practice and mindfulness (Cobb et al., 2015). Another study indicated that individuals who pray regularly and with mindfulness have better mental health than those who do not (Ijaz et al., 2017). Interestingly, in this present study, students with religious affiliation did not show any significant differences in mindfulness and other variables compared to those without a religious affiliation. This may be because the number of adolescents who declared having a faith was too small to have any impact. Moreover, there was a lack of detailed information about the usual religious practices of the participants to explore the possible impacts on well-being.

In summary, there are a few education policy implications from the above study results. Based on the regression analyses, it is evidenced that mindfulness predicted not only stress adversely, but also life satisfaction and life savoring positively. Assessing the level of mindfulness, stress level, life satisfaction, and savoring of adolescents may help screen those students, especially vulnerable groups, for early intervention. According to recent research, mindfulness intervention not only improves the quality of mindfulness and the psychological resilience of adolescents, it also reduces stress by helping students to cope with stress through non-reactive awareness (Liu et al., 2022). Moreover, previous research has shown that mindfulness interventions can also facilitate healthy life habits, mindful eating habits (Hendrickson and Rasmussen, 2017), and mindful social media use (Weaver and Swank, 2019). Developing programs with mindfulness training may help adolescents to enhance their well-being and resilience in challenging times, such as when there are school closures or social distancing policies.

## Limitations and suggestions for further research

Due to the difficulties of school closures during the COVID-19 pandemic, this study was not carried out with stratified sampling. The adolescents from the three schools in this study were mainly from the median level of academic ability in the education system of Hong Kong. Stratified cluster sampling study with students from a diverse range of backgrounds should be considered in the future. Moreover, because of a lack of data regarding socioeconomic status, special education needs (SEN), and underprivileged ethnic minorities, the information on the most disadvantaged adolescents was not articulated for analysis in this study. The above information may be significant for developing school-based mindfulness interventions. From previous research, while various school-based mindfulness programs were effective in enhancing well-being and resilience by significantly reducing stress among adolescents in a Chinese setting (Lau and Hue, 2011; Lam and Seiden, 2020; Schussler et al., 2021; Liu et al., 2022), it is necessary to explore accessible and feasible mindfulness practices targeting the needs of adolescents, especially those of low socioeconomic status and with special needs, during challenging periods such as the COVID-19 pandemic.

In summary, the current study provides encouraging evidence that mindfulness is crucial for enhancing well-being and stress coping among adolescents in a Chinese social context during challenging times. Overall, the findings suggest that researchers and youth service providers may want to explore mindfulness-based training in schools and communities to promote well-being and alleviate the mental suffering of adolescents.

## Data availability statement

The datasets presented in this article are not readily available due to confidentiality. Requests to access the datasets should be directed to ngarszelau@cuhk.edu.hk.

## Ethics statement

The studies involving human participants were reviewed and approved by Human Research Ethics Committee (HREC), The Education University of Hong Kong. Written informed consent from the participants’ legal guardian/next of kin was not required to participate in this study in accordance with the national legislation and the institutional requirements.

## Author contributions

N-sL has contributed to the conception and design of the work, coordination of data collection, and drafting and revising most parts of the work. RC has contributed to the quantitative data analysis, drafting the result section, and revising most parts of the work with critical comment. CL has contributed to data collection and interview. AL has contributed to drafting introduction and some parts of literature review. MF has contributed to exploring literature related to discussion section. All authors contributed to the article and approved the submitted version.

## Conflict of interest

The authors declare that the research was conducted in the absence of any commercial or financial relationships that could be construed as a potential conflict of interest.

## Publisher’s note

All claims expressed in this article are solely those of the authors and do not necessarily represent those of their affiliated organizations, or those of the publisher, the editors and the reviewers. Any product that may be evaluated in this article, or claim that may be made by its manufacturer, is not guaranteed or endorsed by the publisher.
